# Bridging the Gap: Assessing Material Performance of Laboratory Specimens and Concrete Structures

**DOI:** 10.3390/ma16124306

**Published:** 2023-06-10

**Authors:** Juan M. Lozano-Valcarcel, David Ov, Thomas Kränkel, Christoph Gehlen, Rolf Breitenbücher

**Affiliations:** 1 Chair of Materials Science and Testing, Centre for Building Materials, School of Engineering and Design, Technical University of Munich, 80333 Munich, Germany; 2 Institute for Building Materials, Ruhr University Bochum, 44801 Bochum, Germany; david.ov@ruhr-uni-bochum.de (D.O.);

**Keywords:** durability, reinforced concrete, chloride ingress, carbonation, performance testing

## Abstract

Chloride ingress and carbonation pose a significant risk of steel rebar corrosion in concrete structures. Various models exist to simulate the initiation phase of rebar corrosion, addressing both carbonation and chloride ingress mechanisms separately. These models also consider the environmental loads and material resistances, typically determined through laboratory testing based on specific standards. However, recent findings show significant differences between material resistances obtained from standardized laboratory specimens and those extracted from real structures, with the latter exhibiting inferior performance on average. To address this issue, a comparative study was conducted between laboratory specimens and on-site test walls or slabs, all cast using the same concrete batch. This study encompassed five construction sites featuring different concrete compositions. While laboratory specimens adhered to European curing standards, the walls were subjected to formwork curing for a predetermined period (typically 7 days) to simulate practical conditions. In some instances, a portion of the test walls/slabs received only one day of surface curing to emulate inadequate curing conditions. Subsequent testing of compressive strength and resistance to chloride ingress revealed that field specimens exhibited lower material resistance compared to their laboratory counterparts. This trend was also observed in the modulus of elasticity and carbonation rate. Notably, shorter curing periods further compromised performance, particularly resistance to chloride ingress and carbonation. These findings highlight the importance of establishing acceptance criteria not only for concrete delivered to construction sites but also for ensuring the quality of the actual structure.

## 1. Introduction

### 1.1. Background: Change from a Prescriptive to a Performance-Based Design

The durability of concrete and reinforced concrete structures is currently ensured in the relevant codes by means of descriptive requirements, in Europe [1,2,3,4,5,6]. This descriptive approach defines limit values for the concrete composition and a minimum concrete cover in dependence on the expected environmental exposure of the concrete. In the European case, Ref. [2] defines exposure classes for different degrading mechanisms such as carbonation or chloride ingress, for which a minimum cement content, a minimum compressive strength, a maximum permissible water–cement ratio, and a minimum concrete cover have to be met. According to [3], a service life of around 50 years is guaranteed, under normal maintenance measures, if these prescriptive requirements are followed. In contrast, building codes make use of a performance-based approach for assessing the structural performance of concrete by means of partial safety factors (a semi-probabilistic approach) [7,8].

Recently, several investigations have laid the basis for a change from a prescriptive to a performance-based approach. For instance, Refs. [9,10,11] provided a framework to assess the durability of concrete using a full-probabilistic approach, especially in the case of carbonation- and chloride-induced corrosion. References [10,12] highlight that a key advantage of such an approach lies in the possibility of predicting the evolution of the condition state of an element, and [13] provides an example of how to update the prognosis using additional information, such as inspection data. The state-of-the-art and open research questions are presented and discussed in [14,15,16]. Therefore, the change from the current prescriptive approach to a performance-based one in the assessment of the concrete’s durability is a relevant topic. 

In contrast to a prescriptive approach, a performance-based approach is based on three principles: (a) the use of validated models, (b) the quantification of the material resistance (performance) and the acting loads (or exposure), and (c) a safety concept based on a probabilistic basis [10,11]. This contribution focuses exclusively on the quantification of the material resistance. The models provided in [10,11] for assessing the durability of concrete against carbonation- and chloride-induced corrosion require as input the carbonation resistance (estimated under defined accelerated conditions [10,11]) and the chloride migration coefficient (as an alternative to the chloride diffusion coefficient [10]). These tests measure the concrete’s performance against the mentioned mechanisms and are key within a performance-based approach. 

### 1.2. Difference between the Performance of Laboratory Specimens and Specimens Taken from the Structure

Reference [17] states that the quality of concrete produced on the construction site is always lower than the one achieved in the laboratory. Additionally, Ref. [17] mentions that the concrete produced on the construction site is subjected to a considerable quality variation due to variations in concrete production, curing conditions, and workmanship. Moreover, Ref. [18] affirms that some influencing factors that explain these differences include not only the transport, casting, and curing of the concrete on site, as provided in [17], but also local environmental conditions such as temperature, wind speed, solar radiation, and relative humidity, among others. Usually, the quality of concrete in the construction site is mainly assessed by means of separately prepared specimens cast from the concrete “as delivered” to the construction site [2]. Only in exceptional cases are specimens taken directly from the structure to assess the concrete performance [17,18]. By not implementing quality controls on the structure, the performance of the delivered material can be assessed but not that of the actual structure. The latter is, nevertheless, of significant importance for the asset owner [17]. Therefore, following the current building codes, the quality of the concrete “as built” is not being directly assessed, and the effects of casting, compacting, and curing are not directly quantified.

A recent study published by a Swiss research team [18,19] reported that concrete’s performance in real structures differs from that quantified using separate standard specimens, as normally used for assessing the quality of the concrete delivered to the construction site. They cast separate standard specimens from the concrete “as delivered” to the construction site and compared them against specimens taken from the real structure. The results showed that the specimens taken from the real structure had decreased compressive strengths (around 20% lower on average), and increased chloride migration coefficients, as well as higher carbonation rates (50% and 40% higher, respectively) in relation to the separately prepared standard specimens [18,19]. Reference [18] mentions previous studies supporting this hypothesis in [20] for compressive strength and in [21] for durability-related parameters. Therefore, Ref. [18] highlights the importance of implementing quality control measures not only on the delivered material but also on the actual structure. Such affirmation is also mentioned in [17,22].

### 1.3. Quality Control on the Delivered Concrete and the Built Structure

The concrete’s compressive strength is a key parameter for structural design. The chloride penetration resistance, in this case, the chloride migration coefficient, and the carbonation rate are key material parameters for the durability of reinforced concrete structures against chloride-induced and carbonation-induced corrosion, respectively. Therefore, the quantification of these parameters plays a decisive role not only in comparing the performance of materials with different compositions but also within the scope of quality control in reinforced concrete construction.

In the European case, Ref. [2] defines a set of conformity criteria to ensure the quality of the produced concrete up to its delivery to the construction site. Nevertheless, these criteria are strongly focused on the structural performance of concrete, specifically on compressive strength [23] and tensile strength [24]. Additional conformity criteria are defined in [2] for other categories but are mainly directed towards fresh concrete properties, such as the bulk density [25], the fresh concrete consistency (for instance, the spread flow test [26] or the degree of compactability test [27]), and the air content [28], among others. These conformity criteria do not directly cover the concrete’s parameters that are relevant for a performance-based assessment of its durability, such as the carbonation resistance of the chloride penetration resistance. Hence, a different approach is needed. 

Both [17,18] note that carrying out such control measures by extracting cores from the actual structure and performing direct testing on core-drill specimens is not a practicable solution due to the increased effort and costs. A way to circumvent this is by introducing indirect testing methods. A practical example of this was mentioned in [22,29] for the Western Scheldt Tunnel. Here, a probabilistic durability design for concrete subjected to chloride exposure was carried out for tunnel linings, and compliance testing was carried out using the Wenner probe [22,29,30], thanks to a good functional correlation between the electrical resistivity of concrete and the chloride migration coefficient [10,22,29]. Furthermore, Ref. [17] mentions that a similar compliance framework has been implemented in Norway for individual cases. A similar framework is also provided in [31]. 

### 1.4. Focus of the Present Contribution

As mentioned in the previous sections, the quality of the concrete achieved in the actual structure may vary significantly from that of separate standard test specimens. Moreover, Refs. [18,19] highlight the need for further research in this regard. Motivated by this, the authors are assessing the difference between the concrete performance on separate standard test specimens and the actual structure within a German research project. Additionally, some indirect tests are being evaluated within the project for compliance testing in the structure.

This contribution presents the results obtained during the first half of the project and focuses only on the first aspect: the difference between the performance of separate standard test specimens and that of specimens taken from on-site structures. For this purpose, separate standard specimens and test walls or slabs were cast at different construction sites and tested for their compressive strength and chloride penetration resistance using the same concrete batch “as delivered” to the construction site. For a selected number of construction sites, the specimens were also tested for their modulus of elasticity and their carbonation rate under accelerated conditions. The differences between the standard specimens and those taken from the test walls or slabs were compared and analyzed. 

## 2. Materials and Methods

The experimental program focused exclusively on the evaluation of the concrete performance “as delivered” to the construction site. A total of five construction sites, and therefore five concrete mixtures, were evaluated. For each of these concrete mixtures, two main series were tested: the laboratory series and the structure series. The laboratory series was cast using standard laboratory molds and cured under laboratory conditions, whereas, for the structure series, specimens were prepared from drill cores taken from the test walls or from slabs built with the same concrete batch as the laboratory series.

### 2.1. Materials

The concrete of five construction sites was investigated. The construction sites were exclusively bridges located in Germany. The structural elements analyzed were two abutments, two superstructures, and one bridge cap. The technical specifications for these kinds of structures are given in Germany by the ZTV-ING (Zusätzliche Technische Vertragsbedingungen und Richtlinien für Ingenieurbauten; English: Additional Technical Contract Conditions and Guidelines for Civil Engineering Structures) [32], in addition to the Eurocode 2 and the respective national appendix [5,6]. Table 1 shows an overview of the structural elements evaluated and the concrete used for each of them. 

The fresh concrete properties of the delivered concrete were quantified following European standards in accordance with [2]. Table 2 provides a summary of these properties. 

A set of separate standard specimens was prepared from each of the concretes for the laboratory series. For the structure series, a test wall or slab (bridge cap) was built on each of the construction sites by the construction workers, using the same concrete batch and using the methods and tools there available. The specimen preparation and the testing procedures are explained in detail in the next section.

### 2.2. Specimen Preparation and Testing

Usually, the quality of concrete in the construction site is assessed by means of separate specimens cast from the concrete “as delivered” and using standard molds and methods. In order to assess the variation in performance between these specimens and the concrete “as built”, two series were cast and tested following current standards. The first one, the so-called “laboratory” series, was cast from the concrete delivered to the construction site using standard molds and compacted using a vibration table, as it would be done in the laboratory. For the second one, the so-called “structure” series, a test wall (or slab in the case of C3), with dimensions 2.00 m × 2.00 m × 0.25 m, was cast by the construction site workers using the methods and tools there available. Drill cores were later taken from the structure to prepare the test specimens. A test wall was selected for the abutments and the superstructure because the chloride ingress generally takes place at the formwork surface, whereas for the bridge caps, the chloride ingress occurs from the “formwork-free” concrete surface of the concreting (filling) side. 

The laboratory series was cast at the construction site and then brought to the laboratory. The specimens were taken from the molds and placed into water within the first 48 h (mostly 24 h) after casting. They were cured in water afterward until the age of 28 days and subsequently tested. On the other hand, the test walls (C1, C2, C4, and C5) were cured in the formwork for a defined period of time on each construction site, in most cases 7 days, which is standard practice, to simulate the conditions of the “real” structure. The slab (C3) was cured by covering it with a plastic foil. The foil was removed on one half of the slab surface after 1 day, whereas the other half stayed covered for 7 days in total. Table 3 provides the curing times and methods for each of the test structures. Drill cores were afterward taken from the test walls and from the slab before the age of 28 days and subsequently prepared for each test following the corresponding standard.

Since the curing periods between the laboratory and the structure series were different, an additional series (structure*) was, in some cases, considered to bridge this gap. For these series, drill cores were taken from the 7-day curing sections of the test wall or slab directly after the curing was finished (removal of the formwork or the plastic foil), put immediately underwater, and brought to the laboratory. The drill cores were then cured underwater together with the laboratory series and prepared before the age of 28 days for each test. This series constitutes an intermediate case: casting and compaction at the construction site with the methods available there and curing under the same conditions as the laboratory series (except for the first 7 days in the formwork). Table 4 provides an overview of the series considered for each of the concretes analyzed. 

As mentioned previously, in addition to transport, placing, and curing, several environmental factors affect the characteristics of the concrete in the actual structure [17,18]. Some of them include temperature, relative humidity, and solar radiation [18]. Even though some experimental setups provide useful tools to control some of these factors, such as [34], these cannot be controlled at a big scale on the construction site. Thus, this study does not address these factors directly. Table 5 provides the corresponding casting methods, curing, and preconditioning conditions of each of the analyzed series. 

**Figure 1:**
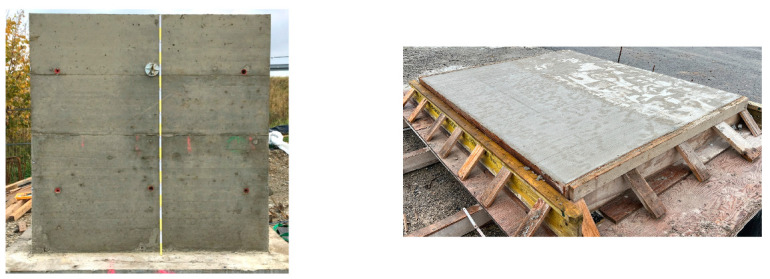
Left: test wall (C4) and right: test slab (C3). Both 2.00 m × 2.00 m × 0.25 m.

Figure 1 shows an exemplarily a test wall (C4) and the test slab (C3).

The prepared specimens were then tested for their compressive strength according to DIN EN 12390-3 [23] and for their chloride penetration resistance by means of the “Rapid Chloride Migration Test” (RCM) according to the German BAW Code of Practice MDCC [35]. Both tests were carried out at the age of 28 days. This test was selected because it delivers the chloride migration coefficient, which is the key concrete performance parameter used for assessing the durability of reinforced concrete against chloride-induced corrosion using a full-probabilistic, performance-based approach according to [10,36]. Additionally, the test can be carried out in a few days, and it provides reproducible test conditions due to a constant medium concentration and voltage application. 

The compressive strength was estimated for the laboratory series using cubes with 150 mm edge length and for the structure series using drill cores with 100 mm diameter and 100 mm height. The RCM test was carried out using cylindrical specimens with 100 mm diameter and 50 mm height, prepared by drilling and sawing of cubes with 150 mm edge length (laboratory series) or from drill cores (structure and structure* series). 

**Figure 2:**
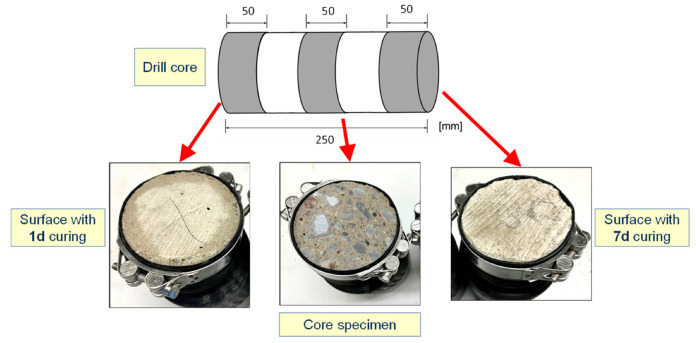
Preparation of the structure series specimens from drill cores for the RCM test.

Since the ingress of chlorides at the structure takes place near the surface of the concrete, the specimens from the structure and structure* series were prepared for the RCM test leaving the “real formwork surface” or “skin” of the concrete as the ingress surface. Additionally, another subseries was prepared using slices prepared from the middle, or core, of the drill core specimen. Figure 2 provides an illustration of the preparation of the specimens from the test structures for the RCM test. The specimens for compressive strength were prepared exclusively from core sections of the drill cores. In contrast, the specimens of the laboratory series were strictly prepared following the corresponding standards.

For cases C4 and C5, specimens were also prepared to analyze their modulus of elasticity and carbonation rate under accelerated conditions. The modulus of elasticity was determined in accordance with [37] (procedure B) at an age of 28 days using cylindrical specimens with 100 mm diameter and 200 mm height. The specimens from the laboratory series were cast using cylindrical molds, whereas the specimens from the structural series were prepared from drill cores exclusively from the core sections. 

The accelerated carbonation rate was determined for C4 and C5 based on [38]. Starting at an age of 28 days, the specimens of all series underwent a preconditioning of two weeks in a laboratory chamber with 20 °C and 65% relative humidity. After that, i.e., at a concrete age of 42 days, the specimens were placed in the testing chamber with controlled temperature (20 ± 2) °C, relative humidity (57 ± 3) %, and a carbon dioxide (CO_2_) concentration of (3.0 ± 0.5) % in volume. Subsequently, the specimens were split and sprayed with a 0.1% (*w*/*v*) phenolphthalein indicator solution after 7, 28, and 70 days of exposure and the carbonation depth was measured [38]. Finally, the accelerated carbonation rate (*K_ac_*) was determined by means of a linear regression using the square root of time approach [38]. This test [38] was selected over a test under natural conditions [39] because it significantly reduces the testing time from 1 to 2 years to only 70 days (excluding curing and preconditioning).

**Figure 3:**
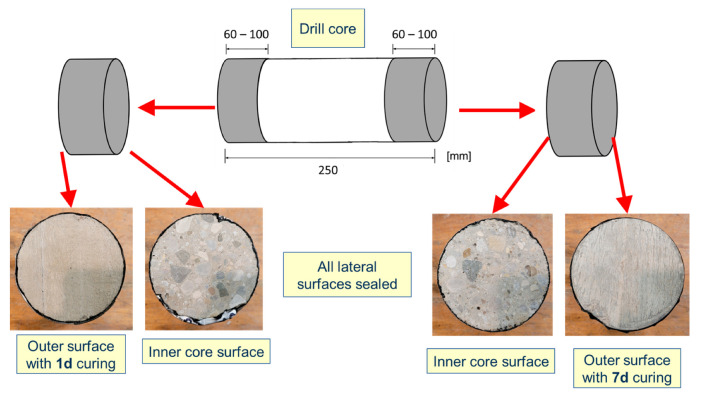
Structure series specimen preparation from drill cores for accelerated carbonation test.

For the laboratory series, prisms with a cross-section of 100 × 100 mm and length of 500 mm were cast, whereas the specimens of the structure series were prepared from the drill cores analogous to the surface specimens for the RCM test. To prevent radial diffusion, the lateral surface of the structural series cylindrical specimens was sealed, leaving two test surfaces: the “outer surface”, or actual concrete surface, and a freshly cut surface, or “inner core surface”. Figure 3 illustrates the specimen preparation for these purposes. The two opposing surfaces of each specimen were considered and analyzed separately. For each testing age, at least two specimens were split and sprayed for each series and the carbonation depth was measured. The length of the specimens varied according to the testing time: for seven days of exposure, around 60 mm; for 28 days, around 80 mm; and for 70 days, around 100 mm.

Not all series could be considered for each construction site due to logistic and time limitations. Table 6 provides an overview of the series cast and tested for compressive strength. Likewise, Table 7 shows the series cast and tested for the chloride migration coefficient.

Finally, Table 8 and Table 9 show the series cast and tested for moduli of elasticity and accelerated carbonation rates, respectively.

## 3. Results

In the present subsection, the results of the experimental program are presented in graphical form. Each of the graphics shows the structural series on the vertical axis and the laboratory series on the horizontal axis. The mean values of each series are provided together with the related standard deviation. Additional lines corresponding to fixed percentage variations are also drawn for a better understanding.

**Figure 4:**
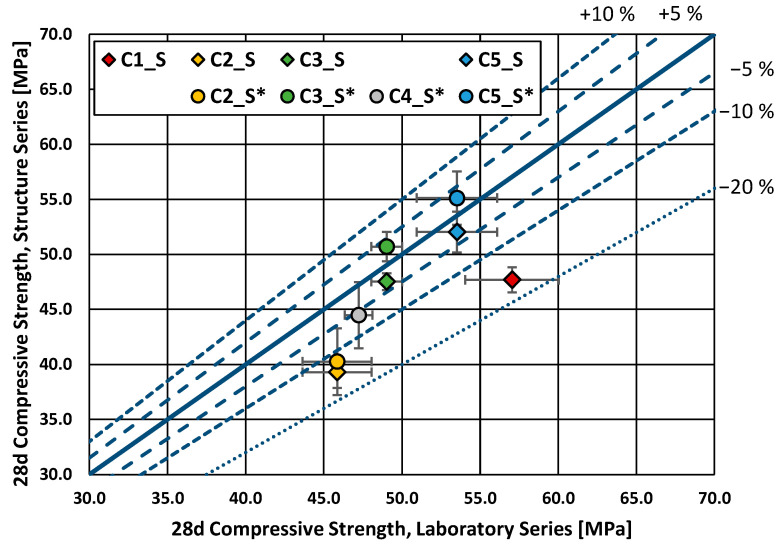
Compressive strength, structure series vs. laboratory series (28 d).

The compressive strength of the structure series was lower than that of the laboratory series. A difference of up to 17% was found for the series C1_S. In contrast, two structure* series performed slightly better (by around 3%) on average than the laboratory series: C3_S* and C5_S*. This difference may be explained by the test accuracy or data scatter since only three specimens were tested for each series. The compressive strength of the structure series was, on average, 10% lower than that of the laboratory series, whereas the compressive strength of the structure* series was only around 3% lower on average than the laboratory series. The compressive strength of the laboratory series reflected the compressive strength class stated in the delivery documents of each concrete. Figure 4 provides the results of the compressive strength test for all the analyzed series.

**Figure 5:**
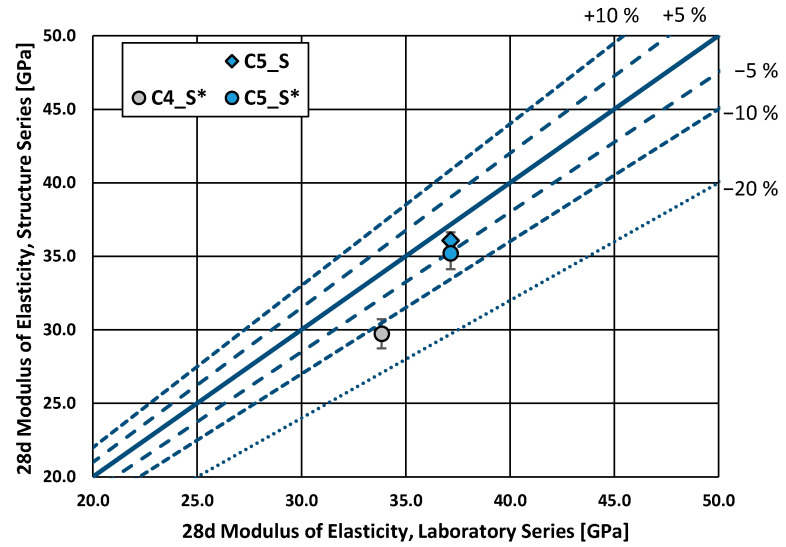
Modulus of elasticity, structure series vs. laboratory series (28 d).

In the test for the modulus of elasticity (C4 and C5, both CEM III/A), the structure series performed worse on average, although only two series were tested (see Figure 5). Different compaction methods, as well as curing conditions, may explain these differences. The values ranged between 30 and 37 GPa, which correspond to values expected from normal concrete.

**Figure 6:**
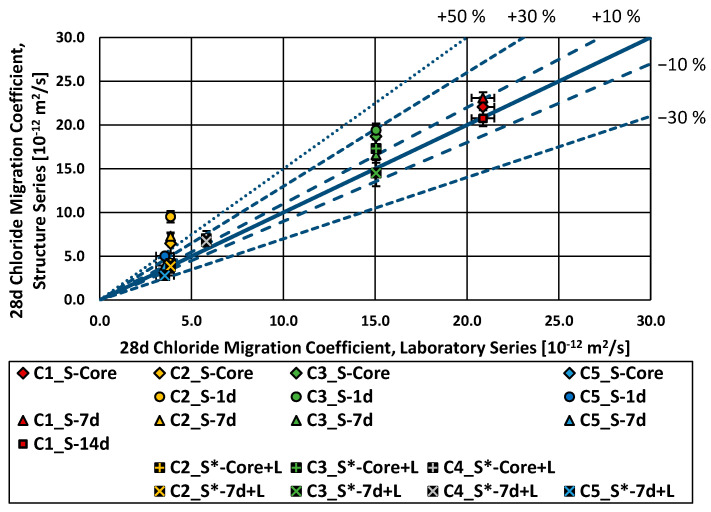
Chloride migration coefficient (28 d), structure series vs. laboratory series.

**Figure 7:**
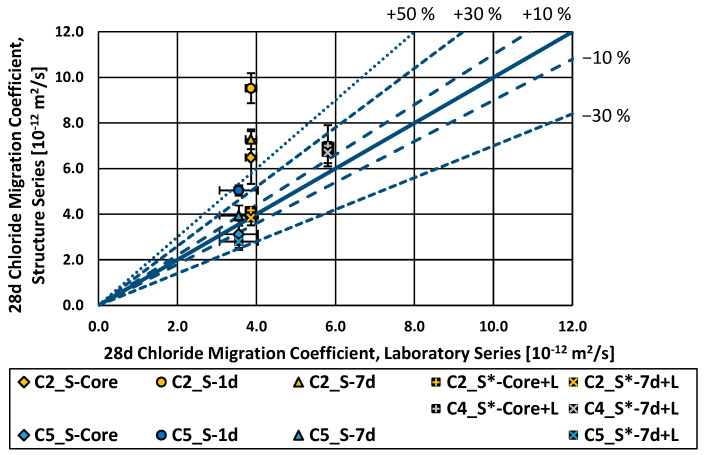
Chloride migration coefficient (28 d), structure series vs. laboratory series, zoom in C2, C4 and C5.

Figure 6 presents the results of the rapid chloride migration test. Figure 7 provides a detailed view of the lower left corner of Figure 6. The chloride migration coefficients of the “S-Core” and “S-7d” series were, on average, 16% higher (worse) in relation to the laboratory series; the “S-1d” series, 50% higher (worse) on average; the “S*-Core” series, around 14% higher (worse) on average. Only the series “S-14 d” and “S*-7 d + L” had a performance comparable to the laboratory series. The effect of curing can be clearly seen, as the performance of the test series improves with longer curing periods. The chloride migration coefficient values of the laboratory series fell in ranges similar to the ones found in [36] for the concrete composition (cement type and w/c-ratio).

**Figure 8:**
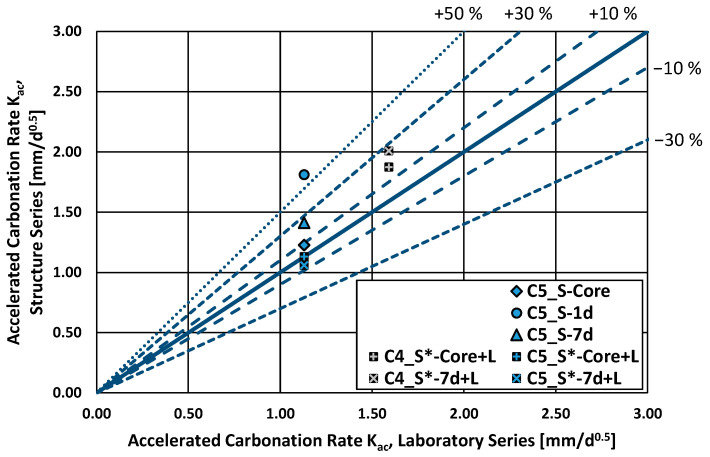
Accelerated carbonation rate (3% CO_2_), structure series vs. laboratory series.

Finally, the results of the accelerated carbonation test (3% CO_2_) are provided in Figure 8. Here, the structural series showed higher carbonation rates than the laboratory series. Furthermore, the effect of curing can be seen in the results of the C5 concrete (CEM III/A). Here, specimens from the structure series cured for only 1 day showed a carbonation rate over 50% higher on average than that of the reference laboratory series. The structure specimens with a curing period of 7 days showed, in comparison, a carbonation rate around 25% higher than the laboratory series, whereas the core subseries (away from the outer areas) showed a carbonation rate around 9% higher than the laboratory series. The C4 (also CEM III/A) structure* series showed a carbonation rate 18% and 26% higher than that of the laboratory series, while the C5 structure* series showed, in contrast, similar results as the reference laboratory series. Nonetheless, the number of series cast and analyzed was limited, highlighting the need for further research, especially considering concretes with a different type of cement.

## 4. Discussion

The results show that concrete cast and cured on construction site, here assessed using test walls and slabs, performs on average worse than separately produced laboratory specimens in compressive strength and rapid chloride migration tests. This inferior performance of the structural specimens compared to the laboratory specimens was also observed at the modulus of elasticity and the carbonation test under accelerated conditions in a limited number of series. These results confirm the findings made in [18,19] and support the hypothesis made by [17].

Nevertheless, previous works [17,18] explain this disparity due to different placement, compacting, and curing techniques between the laboratory and structural specimens. While some European norms prescribe a curing period of 28 days underwater [38], curing is usually carried out on the construction site by leaving the concrete in the formwork or by implementing other curing methods. In the present study, the test structures were cured by leaving them in the formwork (walls) or covering them with plastic foil (slab) and for a shorter period of time (1, 7, or 14 days), simulating usual practice at the construction site (7 or 14 days) and simulating a case with inadequate curing conditions (1 day). Whereas the specimens with insufficient curing (1 day) showed the worst performance, the specimens cured for 7 days showed a better performance but were still inferior relative to the laboratory specimens.

To bridge the gap between the different curing conditions, the series “structure*” was introduced. In this series, the specimens were taken from the test walls or slabs directly after the removal of the formwork and were placed immediately underwater (laboratory conditions). The specimens prepared in this way performed, on average, better than those of the “structure” series cured on site. This suggests that the difference between the performance of the laboratory specimens and the specimens taken from the test structures may be partly explained by the different curing conditions. Nonetheless, the influence of the compaction method and of other variables, such as the climate conditions on site, was not systematically assessed.

Specifically, the compressive strength of the structure specimens was, on average, 10% lower than that of the laboratory specimens. Nonetheless, the structural specimens cured under laboratory conditions showed compressive strengths only 3% lower on average than the laboratory specimens. This suggests that the concrete at the structure may achieve higher compressive strengths if the curing is improved. The modulus of elasticity of the structure series was, on average, lower than that of the laboratory series for two concretes analyzed (both CEM III/A). Nonetheless, the modulus of elasticity was only assessed on two construction sites. Further testing should be carried out to provide more data for a more detailed analysis.

Additionally, a longer curing period improved the performance of the structure specimens in the RCM test on average. The specimens cured for just one day showed a 50% higher (worse) chloride migration coefficient on average than the laboratory series as reference. When the curing time was increased to seven days, the difference in the chloride migration coefficient was significantly reduced. However, the structure specimens still showed a chloride migration coefficient around 17% higher (and thus worse) compared to the laboratory specimens. The difference was further reduced on average to 14% for the structure specimens cured under laboratory conditions (structure*). The only series with a curing duration of 14 days on site showed an average chloride migration coefficient almost similar to the reference (laboratory) series.

Similar results were observed in the accelerated carbonation test. Specimens from one analyzed test structure cured for just one day showed carbonation rates 50% higher (and thus worse) on average than the reference laboratory series. An increase of the curing time to seven days reduced this difference to 25%, whereas the inner core material showed only a difference of 10% in relation to the reference laboratory series. Nonetheless, only a limited number of specimens were analyzed.

The results of the experimental program match the results provided in [18,19]. Furthermore, the controlled curing conditions on the test walls and slabs provided evidence indicating that curing plays a significant role in developing better resistance against chloride ingress and carbonation at the actual structure. Moreover, the results highlight the importance of not only testing the concrete “as delivered” to the construction site but also to assess the performance of the concrete “as built” in the structure. This may be carried out directly by taking drill cores or by means of indirect, non-destructive testing methods [22,31].

However, other concrete properties, such as the resistance to frost–thaw cycles, were not addressed in this contribution but surely would enrich the discussion around this topic. In addition, the influence of concrete mix parameters, such as the cement/binder type, the water/cement ratio, and/or cement content, were not systematically assessed in the present work. This limitation emphasizes the need for further data and similar projects in the future. Furthermore, this study did not consider additional concrete characterization tests that would help explain the disparities observed between the laboratory specimens and those taken from the test structures, such as measuring the real and apparent density of the hardened concrete and its porosity (especially near the outer surface of the structural specimens).

## 5. Conclusions

The experimental program of the present work evaluated the performance of concrete delivered to different construction sites. Five construction sites in Germany, specifically bridges, were studied, and two series of concrete mixtures were tested for each site: the laboratory series and the structure series. The laboratory series involved casting specimens using standard molds and curing them under laboratory conditions. The structure series used drill cores taken from test walls or slabs built with the same concrete batch as the laboratory series. The specimen preparation involved casting separate standard specimens for the laboratory series and building test walls or slabs for the structure series. The curing methods varied, with the laboratory series specimens cured in water for 28 days following European norms, while the test walls and slabs were cured in formwork on the construction sites for specific periods (mostly 7 days) to simulate practical conditions. An additional shorter curing period of 1 day was considered in some cases to simulate inadequate curing. Moreover, additional series were considered in some construction sites to bridge the difference in curing periods between the laboratory and structure series. In this series, the specimens were taken from the test structures directly after the formwork was taken and directly put underwater. The specimens were then tested for compressive strength and chloride penetration resistance using standardized tests. In some cases, the modulus of elasticity and accelerated carbonation rate were also analyzed.

The results of the experimental program indicate that concrete cast and cured on construction sites, as assessed through test walls and slabs, generally performs worse in terms of compressive strength, modulus of elasticity, rapid chloride migration, and accelerated carbonation tests compared to separately produced laboratory specimens. The inferior performance of the structural specimens can be attributed to different placement, compaction, and curing techniques employed on construction sites compared to laboratory conditions. The compressive strength of the structural specimens was, on average, 10% lower than that of the laboratory specimens. The modulus of elasticity was analyzed only on two construction sites. There, the structural series showed lower values for the modulus of elasticity than that of the laboratory series. Moreover, longer curing periods led to improved performance in the rapid chloride migration test. Specimens cured for one day exhibited a 50% higher chloride migration coefficient compared to the laboratory series and, thus, showed an inferior performance. However, specimens cured for seven days showed a reduced difference, although still around 17% higher (poorer performance) than the laboratory specimens. Similar trends were observed in the accelerated carbonation test, with shorter curing periods resulting in higher carbonation rates and, therefore, an inferior performance.

The results of this contribution highlight the importance of curing conditions in developing resistance against chloride ingress and carbonation in concrete structures. This study also emphasizes the need to assess concrete performance not only upon delivery to the construction site but also in the actual built structure. This may be carried out using indirect methods. Examples of such indirect quality testing using non-destructive methods were presented in [17,22,29,31] for the case of the chloride migration coefficient and the Wenner probe (a device for measurement of the electrical resistivity of the concrete). The authors are currently evaluating similar methodologies for assessing the quality control of built structures during the construction phase within the same research project.

Nonetheless, further research is required to explore the influence of compaction methods, climate conditions, and other variables. Additionally, this study did not address frost–thaw resistance or systematically varied concrete mix parameters. Further investigations are needed to provide more comprehensive data and understanding in this field.

## Figures and Tables

**Table 1:** Analyzed structural elements and corresponding concrete mix design as stated in the delivery documents.

Element (Concrete)	Element Type	Strength Class	Cement	w/c-Ratio
E1 (C1)	Abutment	C30/37	CEM II/A-LL	0.49
E2 (C2)	Superstructure	C35/45	CEM III/A	0.45
E3 (C3)	Bridge Cap	C25/30	CEM I	0.48
E4 (C4)	Abutment	C30/37	CEM III/A	0.50
E5 (C5)	Superstructure	C40/50	CEM III/A	0.45
Standard	-	[2]	[33]	-

**Table 2:** Fresh concrete properties of the analyzed concretes “as delivered” to the construction site.

Concrete	Fresh Concrete Temperature [°C]	Spread in Flow Table Test [mm]	Bulk Density [kg/m^3^]
C1	13.6	460	2400
C2	25.2	460	2380
C3	11.0	480	2300
C4	17.8	480	2340
C5	25.6	460	2370
Standard	-	[26]	[25]

**Table 3:** Structural test structures and curing periods for each of the analyzed concretes.

Concrete	Actual Structure	Test Structure Type	Curing Periods	Curing Method
C1	Abutment	Wall	7 and 14 days	In formwork
C2	Superstructure	Wall	1 and 7 days	In formwork
C3	Bridge Cap	Slab	1 and 7 days	Covered with foil
C4	Abutment	Wall	7 days	In formwork
C5	Superstructure	Wall	1 and 7 days	In formwork

**Table 4:** Structural test structures, series analyzed, and tests carried out.

Concrete	Test Structure Type	Series	Tested for ^1^
C1	Wall	Laboratory and Structure	CS and RCM
C2	Wall	Laboratory, Structure and Structure*	CS and RCM
C3	Slab	Laboratory, Structure and Structure*	CS and RCM
C4	Wall	Laboratory and Structure*	CS, RCM, EM, and aC
C5	Wall	Laboratory, Structure and Structure*	CS, RCM, EM, and aC

^1^ CS: compressive strength; RCM: rapid chloride migration; EM: modulus of elasticity; aC: accelerated carbonation.

**Table 5:** Casting, curing, and preconditioning of the test specimens of each series.

Series (Designation)	Cast Tools and Methods	Storage in Mold	Curing
Laboratory (L)	Standard cube molds and vibrating table	1 day	27 days underwater
Structure (S)	Formwork and compaction as “real structure”	-	In formwork for a defined number of days (see Table 3)
Structure* (S*)	Formwork and compaction as “real structure”	-	7 days in formwork, then 21 days underwater

**Table 6:** Series cast for compressive strength test at 28 d.

Concrete	Series		
	Laboratory	Structure	Structure*
C1	C1_L	C1_S	-
C2	C2_L	C2_S	C2_S*
C3	C3_L	C3_S	C3_S*
C4	C4_L	-	C4_S*
C5	C5_L	C5_S	C5_S*

**Table 7:** Series cast for RCM test at 28 d.

Concrete	Series						
	Laboratory	Structure				Structure*	
		1 d ^1^	7 d ^1^	14 d ^1^	Core	7 d + L ^2^	Core + L
C1	C1_L	-	C1_S-7d	C1_S-14d	C1_S-C	-	-
C2	C2_L	C2_S-1d	C2_S-7d	-	C2_S-C	C2_S*-7d	C2_S*-C
C3	C3_L	C3_S-1d	C3_S-7d	-	C3_S-C	C3_S*-7d	C3_S*-C
C4	C4_L	-	-	-	-	C4_S*-7d	C4_S*-C
C5	C5_L	C5_S-1d	C5_S-7d	-	C5_S-C	C5_S*-7d	-

^1^ Curing period on site. ^2^ Curing period on site and placed directly underwater afterward in the laboratory.

**Table 8:** Series cast for the modulus of elasticity test at 28 d.

Concrete	Series		
	Laboratory	Structure	Structure*
C4	C4_L	-	C4_S*
C5	C5_L	C5_S	C5_S*

**Table 9:** Series cast for the accelerated carbonation test.

Concrete	Series						
	Laboratory	Structure				Structure*	
		1 d ^1^	7 d ^1^	14 d ^1^	Core	7d + L ^2^	Core + L
C4	C4_L	-	-	-	-	C4_S*-7d	C4_S*-C
C5	C5_L	C5_S-1d	C5_S-7d	-	C5_S-C	C5_S*-7d	C5_S*-C

^1^ Curing period on site. ^2^ Curing period on site and placed directly underwater afterward in the laboratory.

## Data Availability

Data are available upon request.
